# Assessing parameter identifiability for dynamic causal modeling of fMRI data

**DOI:** 10.3389/fnins.2015.00043

**Published:** 2015-02-20

**Authors:** Carolin Arand, Elisa Scheller, Benjamin Seeber, Jens Timmer, Stefan Klöppel, Björn Schelter

**Affiliations:** ^1^Center for Data Analysis and Modelling (FDM), University of FreiburgFreiburg, Germany; ^2^Department of Physics, University of FreiburgFreiburg, Germany; ^3^Department of Radiology, Medical Physics, University Medical Center FreiburgFreiburg, Germany; ^4^Department of Psychiatry and Psychotherapy, University Medical Center FreiburgFreiburg, Germany; ^5^Freiburg Brain Imaging Center, Departments of Neurology and Psychiatry, University Medical Center FreiburgFreiburg, Germany; ^6^Laboratory for Biological and Personality Psychology, Department of Psychology, University of FreiburgFreiburg, Germany; ^7^BIOSS Center for Biological Signaling Studies, University of FreiburgFreiburg, Germany; ^8^Department of Neurology, University Medical Center FreiburgFreiburg, Germany; ^9^Institute for Complex Systems and Mathematical Biology, King's College, University of AberdeenAberdeen, UK

**Keywords:** functional magnetic resonance imaging, model parameters, dynamic causal modeling, parameter identifiability, profile likelihood

## Abstract

Deterministic dynamic causal modeling (DCM) for fMRI data is a sophisticated approach to analyse effective connectivity in terms of directed interactions between brain regions of interest. To date it is difficult to know if acquired fMRI data will yield precise estimation of DCM parameters. Focusing on parameter identifiability, an important prerequisite for research questions on directed connectivity, we present an approach inferring if parameters of an envisaged DCM are identifiable based on information from fMRI data. With the freely available “attention to motion” dataset, we investigate identifiability of two DCMs and show how different imaging specifications impact on identifiability. We used the profile likelihood, which has successfully been applied in systems biology, to assess the identifiability of parameters in a DCM with specified scanning parameters. Parameters are identifiable when minima of the profile likelihood as well as finite confidence intervals for the parameters exist. Intermediate epoch duration, shorter TR and longer session duration generally increased the information content in the data and thus improved identifiability. Irrespective of biological factors such as size and location of a region, attention should be paid to densely interconnected regions in a DCM, as those seem to be prone to non-identifiability. Our approach, available in the DCMident toolbox, enables to judge if the parameters of an envisaged DCM are sufficiently determined by underlying data without priors as opposed to primarily reflecting the Bayesian priors in a SPM–DCM. Assessments with the DCMident toolbox prior to a study will lead to improved identifiability of the parameters and thus might prevent suboptimal data acquisition. Thus, the toolbox can be used as a preprocessing step to provide immediate statements on parameter identifiability.

## Introduction

Connectivity analyses of fMRI data are noninvasive tools to investigate interactions within a network of brain regions (Smith, 2012). Application of connectivity analysis methods has become more prevalent in a clinical context as diseases are being conceptualized as network disorders (Rowe, 2010; Seghier et al., 2010; Grefkes and Fink, 2014) and it has also become more feasible, as a wide variety of connectivity techniques along with their implementation in toolboxes is available. On the one hand, functional connectivity in task or resting state fMRI data (Biswal et al., 1995, 2010; Lowe et al., 1998; Van de Ven et al., 2004; Calhoun and Adali, 2012) can be investigated e.g., by correlating the time series of activated regions, though interpretation toward causality is limited here (Stephan, 2004). On the other hand, the application of effective connectivity methods such as Dynamic Causal Modeling (DCM; Friston et al., 2003) provides insights into the causality of interactions between certain brain areas (Friston, 2011; Stephan and Roebroeck, 2012).

As the assessment of effective connectivity using DCM becomes more and more widespread (Friston, 2011), the publication of guidelines for DCM (Stephan et al., 2010; Kahan and Foltynie, 2013) has greatly facilitated its employment—for application-oriented studies, see e.g., (Rowe et al., 2010; Deserno et al., 2012; Scheller et al., 2013 and Seghier et al., 2010), for a review of patient studies. Nevertheless, it is difficult to determine if a given research question can at least in principal be answered using DCM prior to actual data acquisition.

DCM-related research questions either encompass inference on model structure or inference on model parameters (Stephan et al., 2010). The former is implemented by the comparison of two or more models involving the same brain regions but different input or connection parameters using Bayesian model selection (Penny et al., 2004; Stephan et al., 2009; Rigoux et al., 2014). Inference on model structure, i.e., discrimination between models, can be optimized by manipulating e.g., epoch durations in an fMRI paradigm (Daunizeau et al., 2011b). To this end, Daunizeau and colleagues employed the Laplace-Chernoff risk to evaluate the lower and upper bound on the model selection error with the aim to minimize this error by choosing an optimal experimental design. They found the optimal experimental design to depend on a given research question with optimal epoch durations being shorter to quantify a feedback connection compared to the optimal durations for a modulatory input.

Apart from model structure, research questions on effective connectivity often concern model parameters *per se*, e.g., the directed connection strength between two regions of interest in the DCM framework. Such questions are frequently of interest if one seeks to determine specific differences in connectivity between a diseased and a control group (Seghier et al., 2010). To reliably answer such questions, it is important to ensure that all model parameters are identifiable, i.e., that the parameters can be estimated unambiguously based on the data (Raue et al., 2009). Thus far, parameter identifiability of DCMs for fMRI data has not been investigated. Therefore, we will introduce identifiability as a benchmark by translating an assessment of identifiability from systems biology to MRI modeling. This specific approach exploits the so-called *profile likelihood* (Raue et al., 2009; Kreutz et al., 2013) that detects identifiable and non-identifiable parameters and provides confidence intervals on the parameter value. Thus, identifiability investigated with the profile likelihood explicates how exact DCM parameters are determined by the amount and quality of fMRI data. We introduce the DCMident toolbox that operates on the profile likelihood and provides such statements on identifiability. The toolbox verifies that parameters are uniquely defined and can be estimated with high accuracy.

In the remaining sections of this report, we will introduce the concept of parameter identifiability together with its importance for DCM. In addition, we will show the functionality of the DCMident toolbox that provides easy-to-use visual output on parameter identifiability using the profile likelihood. Moreover, we will examine non-identifiability with its impact on research questions and provide suggestions to resolve non-identifiability issues. For demonstration purposes, we will use the freely available “attention to visual motion” dataset, which has been studied several times within the DCM for fMRI framework.

## Methods

### DCM for fMRI

Studying effective connectivity in a network of brain areas across different experimental conditions is methodologically challenging. DCM for fMRI describes the biophysical nature of directed interactions between brain areas (Friston et al., 2003) by incorporating two forward models, one at the neuronal and one at the hemodynamic level. A number of introductory articles are available (Friston, 2009; Seghier et al., 2010; Stephan et al., 2010) and the physiological basis of the approach is constantly being evaluated (David et al., 2008; Daunizeau et al., 2011a).

In DCM for fMRI the neuronal states are modeled by a non-linear ordinary differential equation (ODE) (Stephan et al., 2008)

$$\dot{Z}(t)=f(Z(t),u,\theta )=\left(A+\sum_{i=1}^{m}B^{\left(i\right)}u_{i}+\sum_{j=1}^{n}Z_{j}D^{\left(j\right)}\right)Z+Cu,$$

where ***Z*** = [*Z*_1_, …, *Z_m_*]^*T*^ is a vector composed of the *m* neuronal activities *Z_i_* in the different brain regions, ***u*** are the stimuli and θ = [θ_1_, …, θ_*n*_]^*T*^ the parameters. This nonlinear state equation describes the change of activity in the brain due to exogenous inputs and modulations. For most applications the bilinear state equation without the nonlinear term, i.e., ***D*** = 0, is used (Friston et al., 2003; Stephan et al., 2008).

This bilinear equation contains three sets of model parameters predefined by the user: First, input parameters, specified in the ***C***-Matrix of the state equation, determine how experimental stimuli enter the model. Second, assumptions about the fixed connections (***A***-Matrix), which represent average connection strengths over task conditions, are specified. A third set of modulatory parameters (***B***-Matrix) expresses expected changes in connection strengths caused by the applied experimental conditions. The connection strength between regions is reported as rates of change in Hz, while a negative value is interpreted as decreased and a positive value as increased coupling from one region to another. With our approach, we will be able to judge whether all these parameters can be correctly estimated.

The neuronal states specified in the equation cannot be observed directly, only the induced BOLD response is measurable. The BOLD response is a convolution of the neuronal states with the hemodynamic response function. This is modeled by the balloon-windkessel model (Friston et al., 2000) which describes the change of vasodilatory signal in brain region *i* due to neuronal activity *Z_i_*. The increased activity leads to a local increase in oxyhemoglobin content *q_i_*. The change of vasodilatory signal itself changes the inflow *f_i_* which leads to changes of blood volume ν_*i*_. The BOLD-signal is finally given by a nonlinear function ***Y*** = λ(*q*, ν), which depends on the oxyhemoglobin content q and the blood volume ν (Friston et al., 2003; Obata et al., 2004; Stephan et al., 2007b). The convolution of the neuronal states with the hemodynamic model can be simplified by using an approximation to the hemodynamic response. This approximation is gained by using two Gamma probability density functions:

$$\Gamma_{pdf}(h,l,t)=\frac{l^{h}t^{\left(h-l\right)}\text{exp}(-lt)}{\Gamma (h)},$$

where Γ(*h*) is the Gamma function. Here, *h*, the shape parameter, with *h* > 0, determines the maximum of the distribution and *l* as the scale parameter with *l* > 0 the elongation of the distribution. The canonical hemodynamic response function (HRF) is a typical BOLD impulse response. It is parameterized by a peak delay of 6 s and an undershoot delay of 16 s. The peak-undershoot ratio is one to six (Friston et al., 2007). Thus, the canonical HRF is given by:

$$HRF(t)=\Gamma_{pdf}(6,1,t)-\frac{1}{6}\Gamma_{pdf}(16,1,t).$$

The observations are given by the convolution of the neuronal activity with the hemodynamic response function:

$$Y(t)=\int_{0}^{\tau^{\prime }}Z(t-\tau )HRF(t)d\tau .$$

We employed the canonical HRF rather than the full balloon model (Obata et al., 2004; Stephan et al., 2007b) in our profile likelihood based approach to allow for increased computation speed. If needed, the toolbox is open to modifications by users, e.g., to include more complex representations of hemodynamics.

### Parameter estimation

In studies on fMRI effective connectivity, it is of interest to get a quantitative measure of coupling strengths between regions of interest and how much experimental stimuli influence this connectivity. To accomplish this, DCM requires a plausible hypothesis-driven model for the neuronal states. Based on this model, the aim is to estimate the parameters of the neuronal state model ***Z***(*t*) from the measured observations ***Y***(*t*).

SPM–DCM uses a Bayesian approach for parameter estimation (Friston et al., 2003). For our method we chose to use a likelihood-based approach, which is numerically more efficient. The main difference between those two methods is that Bayesian parameter estimation requires priors, which can carry valuable information of certain parameters. Using the profile likelihood approach constitutes the “worst case scenario” addressing the question if the data alone contain enough information to reliably estimate a parameter (Raue et al., 2012). This presents valuable information as the users of DCMident would readily know which of the parameters in the final DCM model need to follow the priors. This enables a much more sensible application of the Bayesian DCM after assessing identifiability. Given the close relation between the profile likelihood and the Bayesian DCM, the concept of profile likelihoods can be transferred to the Bayesian DCM. As this would add considerable numerical complexity, we refrained from the implementation.

To assess the agreement of the model with the data, an objective function is applied. For a least-squares optimization this is usually the weighted sum of squared residuals:

$$\chi^{2}(\theta )=\sum_{i=1}^{m}\sum_{k=1}^{d_{k}}\frac{{\left(Y_{i}(t_{k})-{\hat{Y}}_{i}(t_{k},\theta )\right)}^{2}}{\sigma_{i}}$$

Where *d_k_* denotes the number of data-points, *i* = 1 … *m* the number of observables and *Y_i_* and Ŷ_*i*_ the *i*-th observable and its estimate based on the chosen model, respectively. The σ_*i*_ are the corresponding measurement errors, i.e., the standard deviation.

The parameters are then estimated by minimizing the weighted sum of squared residuals with respect to the parameter vector θ:

$$\hat{\theta }=\underset{\theta }{\min}\left[\chi^{2}(\theta )\right].$$

This is consistent with the maximum likelihood estimation (MLE) of the parameter vector θ for normally distributed measurement noise as:

$$\chi^{2}(\theta )=const-2\log(L(\theta ))$$

where

$$L(\theta )=\prod_{i=1}^{m}\prod_{k=1}^{d_{k}}\frac{exp\left(-\frac{1}{2}{\left(\frac{Y_{i}(t_{k})-{\hat{Y}}_{i}(t_{k})}{\sigma_{ik}}\right)}^{2}\right)}{\sqrt{2\pi \sigma_{ik}^{2}}}$$

is the likelihood function of the parameter vector θ. This is possible because the logarithm is a monotone function. So taking the logarithm does not change the position of the extrema and minimizing χ^2^(θ) corresponds to maximizing the log-likelihood function.

### Parameter identifiability

#### Confidence intervals

By analysing the shape of the likelihood function, it is possible to obtain confidence intervals (CIs) for a confidence level α for the estimated parameters. These CIs depict that the true parameters lie within the intervals [σ^+^, σ^−^]_*j*_ with probability α, where *j* = 1, …, *n* and *n* the length of the parameter vector. Here σ^+^ is the upper bound and σ^−^ the lower bound. The likelihood-based CI can be defined by a threshold Δ_α_ in the likelihood (Meeker and Escobar, 1995):

$$\left\{\theta |\chi^{2}(\theta )-\chi^{2}(\hat{\theta })<\Delta_{\alpha }\right\}\text{ with }\Delta_{\alpha }=Q(\chi_{df}^{2},\alpha ),$$

where χ^2^(θ) is the χ^2^-value of the true parameter vector, i.e., the presumed minimum of the likelihood function, and χ^2^($\hat{\theta }$) is the χ^2^-value of the estimated parameter vector. The threshold Δ_α_ is given by an α-quantile *Q* of the χ^2^-distribution. It represents the point-wise CIs with *df* = 1 (*df* = degrees of freedom).

A widespread method is to use asymptotic CIs based on the covariance matrix (Meeker and Escobar, 1995). These can be derived from the curvature of the likelihood function using a quadratic approximation of χ^2^ at the estimated optimum $\hat{\theta }$ given by the Hessian matrix. Asymptotic CIs are a good approximation of the actual uncertainty of $\hat{\theta }$_*i*_ if the amount of data is large compared to the number of parameters and it is exact if the observables depend linearly on the parameters θ. However, these two prerequisites are often not fulfilled (Joshi et al., 2006). In these cases the quadratic approximation is not fulfilled either and higher order terms cannot be neglected. Asymptotic CIs can always be transformed into symmetric parabolas by reparametrisation. However, when higher order terms cannot be neglected, the actual CIs might not be symmetric (for a comparison between standard CIs and likelihood-based CIs see Cook and Weisberg, 1990). For DCMs the observables depend non-linearly on the parameters due to the convolution with the HRF. Therefore, the assumption that the quadratic approximation is valid is questionable and it cannot be decided a priori if the assumption is right. Hence, asymptotic intervals are not appropriate for the application to DCMs and likelihood-based confidence intervals are used in our approach. Likelihood-based confidence intervals do not assume a quadratic approximation. Therefore, they are superior to asymptotic confidence intervals based on the quadratic approximation of the Fisher information matrix (Meeker and Escobar, 1995). Furthermore, likelihood based CIs are reparametrisation-invariant and thus do not depend on the chosen parametrisation.

#### Identifiability and non-identifiability

Based on the CIs, practical identifiability can be defined (Raue et al., 2009, 2010, 2011): A parameter is practically identifiable if it has finite CIs σ^+^ < ∞ and σ^−^ > −∞. Hence, the parameter can be estimated unambiguously from the data. Practical identifiability depends on overall amount and quality of data, such as suitable signal to noise ratio (SNR) and sufficient data points. Practical non-identifiabilities can be resolved by changing experimental design or imaging specifications, e.g., by acquiring more volumes during the fMRI session, by increasing the frequency of a stimulus of interest or by modifying acquisition parameters such as TR. It may occur that a given parameter is practically identifiable, while its CI includes zero. As a consequence, the connection represented by this parameter is compatible with zero and might not exist. Thus, a different model without the respective parameter or a different experiment might have to be chosen to make a clear statement on the respective connectivity (Raue et al., 2010, 2011).

Identifiability of all parameters is a necessary prerequisite for reliably answering research questions pertaining to the respective parameters (Raue et al., 2009, 2010). Practical non-identifiabilities can be discovered by analysing the likelihood function. They can be resolved by adjusting above-mentioned cornerstones of data acquisition. In total, by analysing the parameter identifiability, the reliability of model predictions can be improved (Raue et al., 2009).

### The DCMident toolbox

#### Derivation of the profile likelihood

To analyse the identifiability of the model parameters, we use the profile likelihood

$$\chi_{PL}^{2}(\theta_{i})=\min_{\;\theta_{j\neq i}}\left[\chi^{2}(\theta )\right].$$

With this approach, a section along the minimum of the objective function with respect to all other parameters θ_*j* ≠ *i*_ is computed for each parameter θ_*i*_ individually. So the *n*-dimensional parameter space, with *n* the number of parameters, is explored for each parameter along the direction of least increase of the χ^2^(θ)-value in the χ^2^(θ)-landscape. This is accomplished by varying the considered parameter θ_*i*_ around the minimum of the negative log likelihood χ^2^(θ) and optimizing the other parameters θ_*j* ≠ *i*_ in order to minimize the objective function. As the profile likelihoods are not mere approximations of the log-likelihood as a quadratic 1-dimensional function but optimizations of all parameters but the fixed one taking into account the whole model structure, the suggested approach based on the profile likelihoods enables a sensible derivation of confidence intervals even in the presence of nonlinear models and correlated parameters. We emphasize that the profile likelihood-based approach leads to the same results as asymptotic CIs if the approximation of the log-likelihood function as a quadratic function is valid.

Based on the profile likelihood practical non-identifiability can be defined anew: The profile likelihood of a practically non-identifiable parameter has a minimum but it never crosses the threshold △_α_ for a desired α confidence level in one or both directions and the CI is infinite. So there can be an upper or lower bound on the CI. If for example an upper bound but no lower bound exists, a higher parameter value than this upper bound is not compatible with the data. However, it cannot be decided which parameter value below the upper bound is the best to fit the data (Raue et al., 2009).

#### Components of the DCMident toolbox

To calculate the profile likelihood, both simulated and real fMRI data can be used. Simulations of models are based on the DCM.mat-file obtained from the DCM extension of the SPM software after DCM specification and estimation (see Chapter 32 in the SPM manual, http://www.fil.ion.ucl.ac.uk/spm/doc/manual.pdf). The required design information, e.g., stimulus characteristics and imaging specifications, is obtained from the DCM.mat file and written into two new files using customized MATLAB-functions (http://www.mathworks.de/products/matlab/). Based upon these files the assessment of the respective DCM is started. It is equally possible to use “customized” models, i.e., models, which are not based on a DCM-estimate: By removing regions or connections from the actually estimated model, it can be determined if a less complex “reduced model” becomes identifiable under current experimental design specifications.

Both for the simulation of the data sets and the derivation of the profile likelihoods, the differential equations which represent the neuronal model under investigation are integrated with time steps of *ts = TR/#slices*. For the exogenous stimuli and modulations, boxcar or stick functions depending on the respective experimental design, are used. Thus, the time series is split into short time bins whenever the input changes, such that the ODE can be solved piecewise. The obtained time series ***Z***(*t*) is then convolved with the HRF composed of the two Γ-pdf-distributions. For data acquisition, Gaussian noise *N*(0, σ_*y*_) with the variance of ***Y***(*t*) with respect to the SNR is added to these deterministic “BOLD”-signals.

To calculate the profile likelihoods of the parameters, all model parameters are estimated first. Starting from these parameter estimates $\hat{\theta }$, which also constitute the minima of the respective profile likelihoods, the profile likelihoods are calculated. This is achieved by systematically changing the respective parameter and reoptimizing all other parameters to gain the least χ^2^(θ)-value, meaning that for calculation of the profile likelihood of parameter θ_*i*_, the parameter θ_*i*_ is varied around the first estimate $\hat{\theta }$_*i*_, θ_*i,new*_ = $\hat{\theta }$_*i*_ ± *d*θ_*i*_.

To optimize parameters, start values are needed. For our analysis we used a slightly changed posterior parameter estimate θ ± 0.1 from model inversion as initial guesses. With real data, the easiest way to define start values is to use the connectivity priors as defined in SPM (http://www.fil.ion.ucl.ac.uk/spm/). DCMs depend sensitively on their parameter values as models with circular connections start oscillating for certain parameter sets. Additionally, DCMs can diverge if the inhibitory self-connections are not strong enough to prevent run-away excitation (Friston et al., 2003). If this happens, the start values must be readjusted by e.g., changing the sign of a few parameters. For the optimization, a Levenberg–Marquardt algorithm is used.

For a proper comparison of different identifiability scenarios of an entire parameter set, a single criterion being straight forward to interpret is needed. As confidence intervals and thus the variance of the parameters are of interest, the mean confidence interval (mCI) of the parameter set can be examined; this is referred to as A-optimality in the respective literature (Chernoff, 1953). As non-identifiable parameters have infinite CIs, the CI is set to infinity; for those parameters that have only a lower or upper bound it is infinity as well.

#### Description of test data

**Figure 1:**
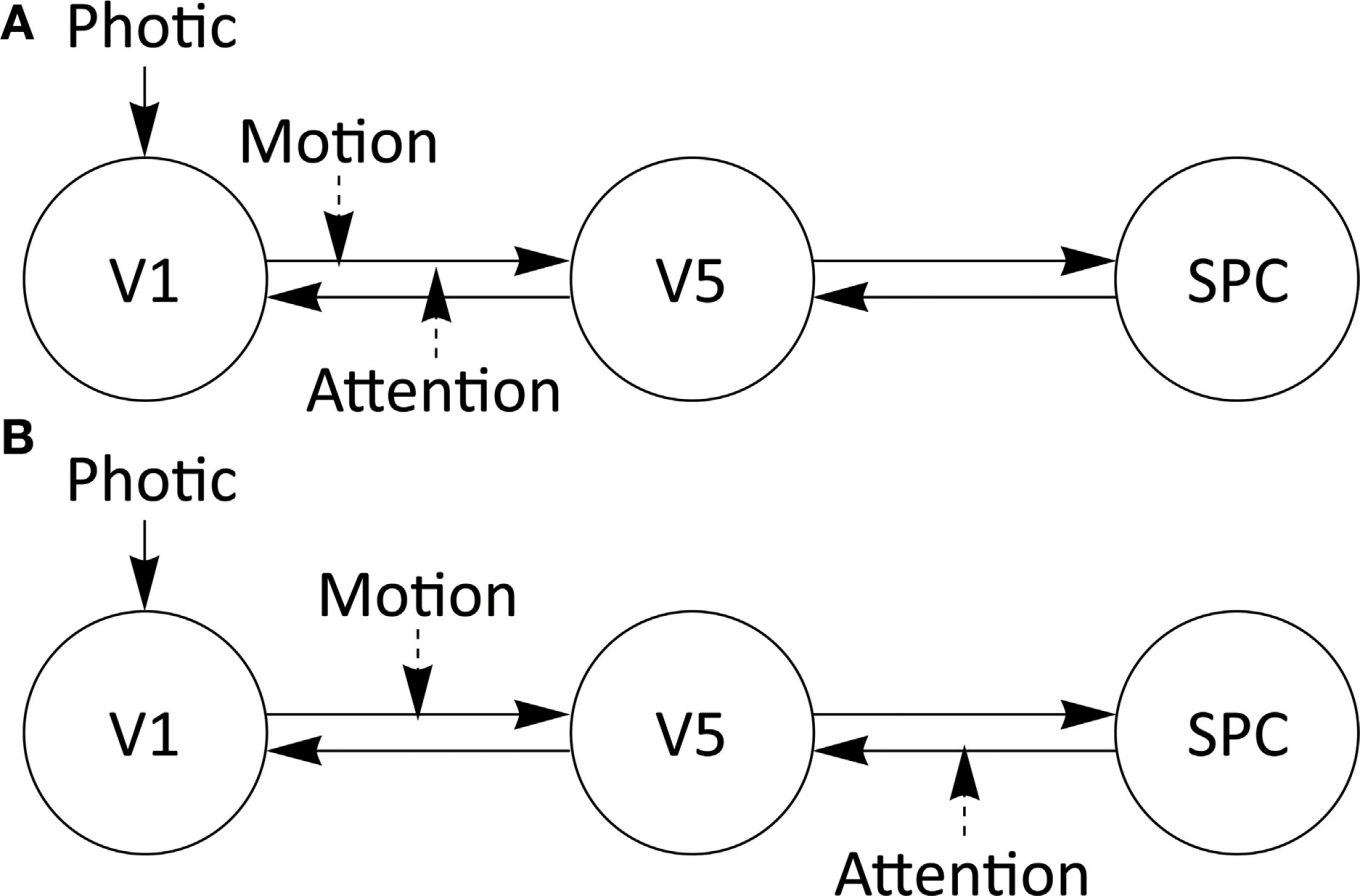
**Three-region models specified for the attention to visual motion dataset**. Driving and modulatory inputs are denoted by Photic and Motion and Attention, respectively. V1, primary visual cortex; V5, extrastriate visual area V5; SPC, superior parietal cortex. **(A)** Attention to motion DCM with experimental modulations on one forward connection (“forward model”) from V1 to V5. **(B)** Attention to motion DCM with experimental modulations on one forward and one backward connection (“backward model”), V1 to V5 and SPC to V5, respectively. For subsequent parameter assessments, the DCM-estimates from the original single-subject dataset were used.

To investigate the performance of our approach, we chose to assess parameter identifiability and the influence of data acquisition adjustments thereon with a three region model specified for the “attention to visual motion” dataset first examined by Büchel and Friston (1997) and Büchel et al. (1998). Please see Büchel and Friston (1997) for a detailed description of task design and experimental manipulations. This dataset is described in several publications and different DCMs to explain the data have been suggested (Penny et al., 2004; Stephan et al., 2004, 2007a; Marreiros et al., 2008; Friston et al., 2011). To analyse the influence of different design parameters, we chose to investigate the “forward model” proposed for this dataset in the SPM manual (see http://www.fil.ion.ucl.ac.uk/spm/doc/manual.pdf) with SPM8 (release 4667) and DCM10. In this three-region model, visual area 1 (V1) and visual area 5 (V5) are bidirectionally connected as are V5 and the superior parietal cortex (SPC). There is one exogenous input (“Photic”) onto region V1 and two modulations (“Attention” and “Motion”), both onto the connection between V1 and V5 (Figure 1A). Additionally, an alternative “backward model” with the same A- and C matrices, but with one modulation onto the connection V1 to V5 and one modulation onto the connection SPC to V5 (Figure 1B), is analyzed for its parameter identifiability.

The testing of identifiability was based on the original imaging specifications: repetition time (TR) of 3.22 s, acquisition of 32 slices per volume and acquisition of 360 volumes in total as well as an epoch duration of 32.2 s. For the SNR, a rather high value of $\sqrt{\mathrm{10}}$ ≈ 3, 2 was chosen to make changes of identifiability due to design or imaging specifications unambiguously apparent. The posterior parameter estimates from the original single-subject dataset after model inversion with DCM 10 in SPM8 (r4667) were used as parameter values for the simulation of the synthetic fMRI data used for the analysis. For the data generation the deterministic model was integrated and afterwards convolved with the canonical HRF to gain the deterministic BOLD-response. Subsequently, random Gaussian noise corresponding to the SNR was added to this deterministic BOLD-response.

To demonstrate the influence of certain imaging specifications on parameter identifiability, we varied TR, number of acquired volumes, i.e., session duration, and epoch duration within certain boundaries typically found in fMRI experiments. Furthermore, an assessment was done with a much lower but more realistic SNR of 1 (Krüger and Glover, 2001). TR was varied between 1 and 3 s in steps of 0.5 s. To additionally demonstrate identifiability changes with long TR values common in older fMRI studies, we also tested a TR of 6.44 s, twice the original TR of 3.22 s. Session duration ranged from 180 to 720 times the TR, i.e., half and twice the duration compared to the original setting, with increments of 90. Epoch duration spanned between 10 and 35 s, with 5 s steps and an original length of 32.2 s. To capture event related design scenarios as well, additional scenarios with shorter epochs were introduced with epoch durations of 1, 3, and 5 s, respectively. In order to gain experimental designs with short epoch durations but the same amount of stimuli, the distance between the stimulus onsets were kept constant, while the length of the stimuli themselves were varied. For each variation of imaging or design specifications we simulated one data set, whereas always a different noise realization was used. Hence, we can demonstrate how parameter identifiability is altered depending on the the sampling rate, the amount of data measured, frequency of stimuli as well as noise. This parallels former work from systems biology manipulating the amount and quality of measured data to gain finite confidence intervals of the profile likelihood and therewith improved parameter identifiability (Raue et al., 2009, 2010).

We expected to find variations in parameter identifiability along these manipulations of imaging specifications. First, we hypothesized that a shorter TR would enhance parameter identifiability because of overall increase in measurement precision (Feinberg and Yacoub, 2012). Second, the acquisition of additional volumes was thought to improve identifiability as well, while a shorter imaging session would lead to a decrease in identifiability, as less data to fit the model would then be available. Third, we hypothesized to find certain epoch durations in block as well as event related design scenarios specific for optimal identifiability. Finally, decreased SNR was considered to diminish overall identifiability.

## Results

### Results of attention to motion “forward” model

**Figure 2:**
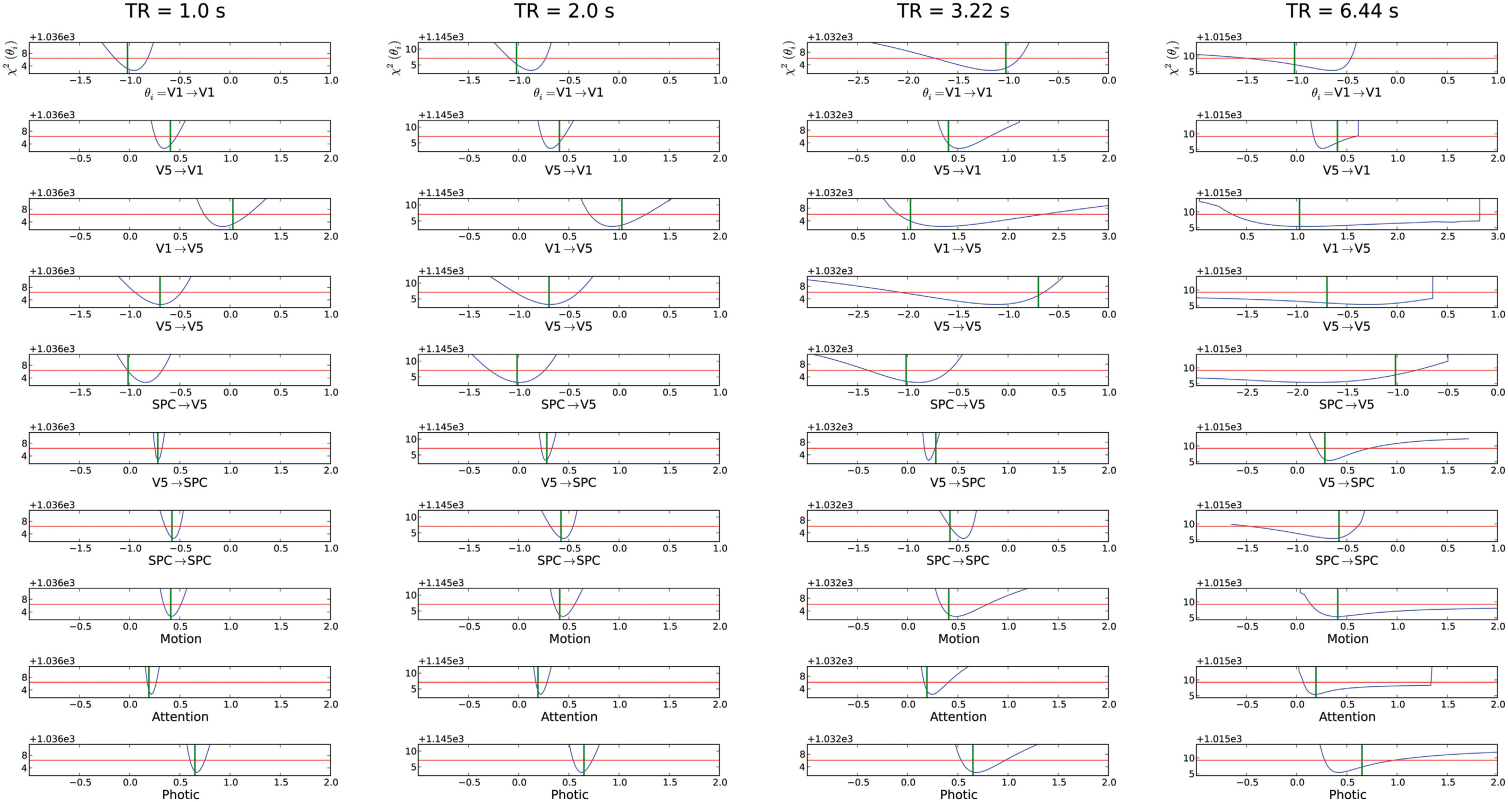
**Assessment of different TRs (1, 2, the original 3.22 and 6.44 s) for the “forward model.”** The parameter value used for the simulation is shown as a green vertical line. The blue line corresponds to the profile likelihood. The red horizontal line illustrates the χ^2^-quantile to a significance level of 1 − α with α = 5%. The parameter is identifiable if the blue line crosses the red line twice, i.e., if boundaries on confidence intervals exist. The respective connection is described below the individual profile likelihood plot with the respective parameter value range. The y-axis depicts the χ^2^-value of the model fit (see definition of χ^2^ in Section “Parameter Estimation”). This value describes the residual information of the data which cannot be explained by the model alone e.g., noise or residual information due to an improper model. Note that x-axis range can change across subplots to enable full depiction of confidence intervals, while the scaling does not change. For the mean confidence interval mCI we gained values 0.22, 0.29, 0.61, ∞ for a TR of 1 to 6.44 s, respectively.

After testing the original imaging specifications of the forward model, all parameters proved identifiable, i.e., showed CI boundaries as depicted by the blue line which crosses the red line twice (Figure 2). Nevertheless, several of these parameter CIs were relatively broad (see e.g., original settings in TR = 3.22 s subplot of Figure 2), implying that the true parameter value cannot be determined with high precision. By shortening TR, testing different epoch durations as well as by increasing session duration, we sought to shrink these CIs to enable more reliable statements about the respective parameters. Effects of such variations are described in the following paragraphs. The whole identifiability assessment could be achieved within reasonable computing times. For the models chosen in this report, we needed 5 h on average for all parameters in a cluster computer environment.

#### Variation of TR

We were able to obtain identifiable parameters across most simulated TR values. Though TR was simulated in 0.5 s increments between 1 and 3.5 s, we chose to depict a subset of changes in parameter identifiability only (Figure 2). Plots of remaining TR values are displayed in Supplementary Material (Supplement 1). As expected, a short TR of e.g., 1 s leads to an overall shrinkage of CIs. Most prominent changes in parameters' CIs could be observed in connections concerning the ***A***-matrix of the DCM state equation. Modulatory as well as exogenous inputs (***B***- and ***C***-matrices) already had relatively narrow CIs in the original setting—see subplots for “Photic,” “Attention,” and “Motion” in Figure 2. When testing TR = 6.44 s, i.e., twice the original TR, we observed non-identifiabilities for the parameters of the connections leading to V5 and also for the modulating exogenous inputs of the ***B***-matrix. Interestingly, the self-connection of V5 is consistent with zero as the profile likelihood approaches the threshold in the negative range on the one side and crosses in the positive range on the other side. Therefore, this connection might not exist. From the point of systems theory, this connection should exist as an inhibitory mechanism to prevent run-away excitability and to model the decay of activity (Friston et al., 2003; Stephan, 2004). Based on this data, the existence and strength of this connection could not be decided when a TR of 6.44 s is chosen. For all other practically non-identifiable parameters, an upper or lower bound excluding zero is apparent. These connections do exist in this scenario, but the parameter values have huge error bars in one direction. For TR = 6.44 s there are parameter regions apparent where the profile likelihood suddenly jumps to very high χ^2^(θ)-values. In these cases a parameter regime is reached where the time series diverges and optimizing the other parameters cannot counterbalance the fixed parameter value.

#### Variation of session duration

**Figure 3:**
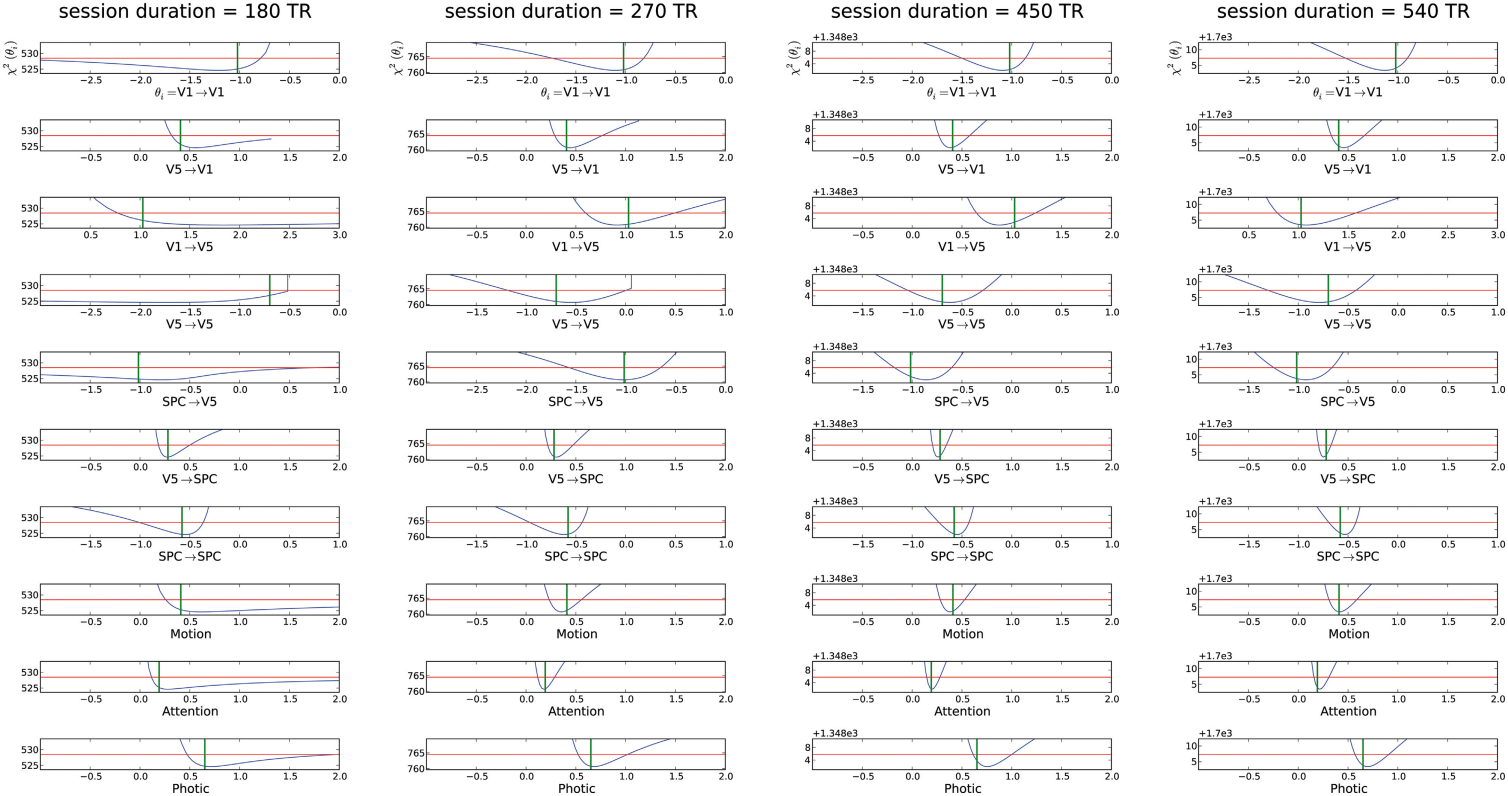
**Assessment of different session durations ranging from 180 to 540 times the original TR for the “forward model.”** For explanation of the graphs see Figure 2. The mCI were from left to right ∞, 0.58, 0.38, 0.40.

Based on the original experimental run of 19.32 min (i.e., 360 acquired volumes), we changed session duration to determine the number of volumes necessary for overall parameter identifiability, while TR and epoch duration kept the values of the original study. As already stated above, all parameters were identifiable in the original setting with 360 acquired volumes, therefore this scenario is not depicted anew (see Figure 2). It is evident from Figure 3 that halving the session duration (i.e., 180 acquired volumes) does not suffice to obtain identifiable DCM parameters. Several CIs either lack an upper or lower bound, i.e., the profile likelihood does not cross the threshold twice (Figure 3). In terms of the above-introduced non-identifiability terms, these are practical non-identifiabilities, which can be resolved by acquiring a sufficient number of volumes, while the envisaged DCM itself does not need to be modified. A change toward identifiability of all parameters can already be observed with 270 volumes. When increasing the number of volumes beyond 360, a further narrowing of CIs on parameter estimates could be observed but not perceivably beyond 540 acquired volumes (please refer to Supplementary Material (Supplement 1) for plots of 630 and 720 acquired volumes). For a session duration of 540 times TR the mean CI even increases slightly, though not considerably.

#### Variation of epoch duration

**Figure 4:**
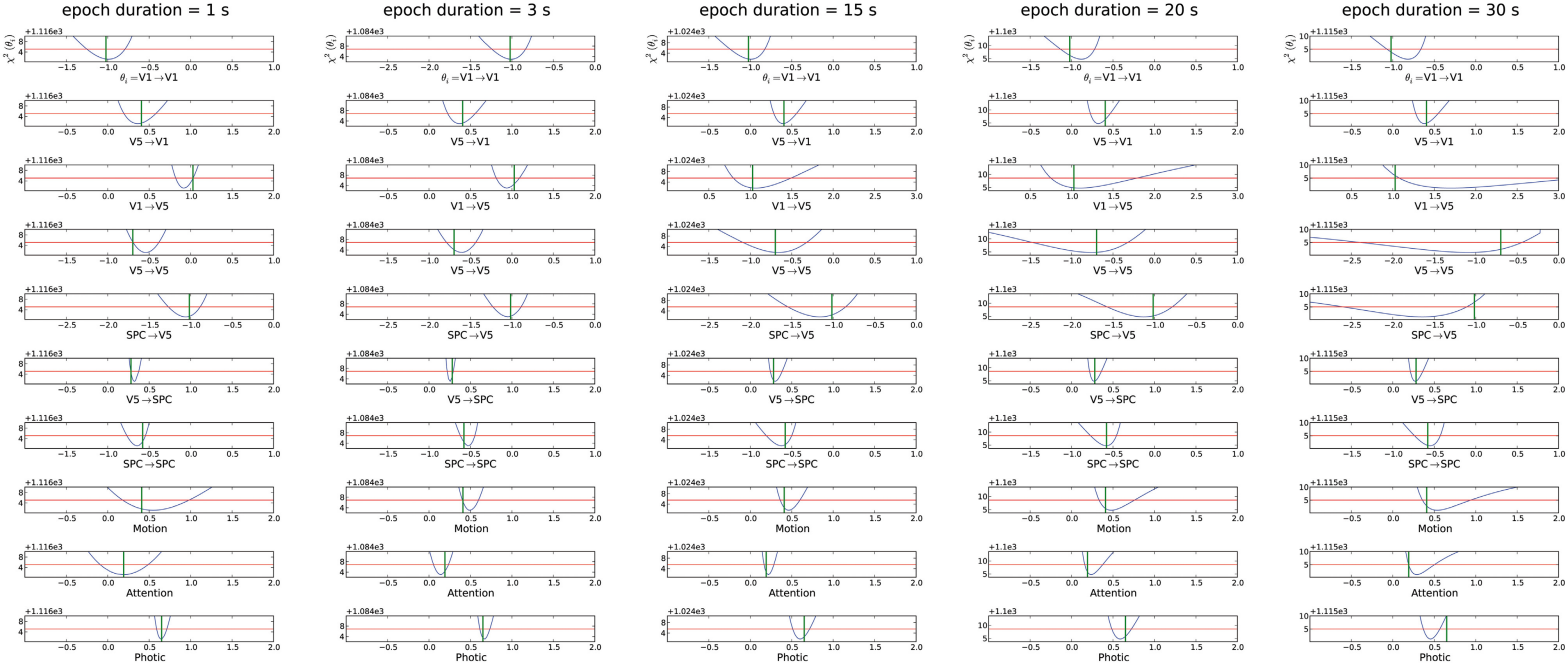
**Assessment of different epoch durations ranging from 1 s, which is equivalent to stimulus durations of rapid event-related designs, up to a blocked design equivalent of 30 s**. The mCI were from left to right 0.32, 0.22, 0.36, 0.46, and 0.72. For explanation of the graphs see Figure 2.

Different epoch durations referring to either event-related or block design scenarios were tested for parameter identifiability characteristics. Potential event-related designs with epoch durations of 1, 3, and 5 s were assessed (see subplot for 1 and 3 s in Figure 4). As the original experiment was a blocked design, the remainder of Figure 4 shows such a scenario with epoch durations of 15, 20, and 30 s, the latter being marginally shorter than the original setting of 32.2 s. As can be concluded from comparing the original setting in the rightmost subplot of Figure 2 to the 30 s subplot of Figure 4, even a small decrease of epoch duration improves parameter identifiability. The results show that for this model and these design parameters, a decrease in epoch duration improves identifiability. Furthermore, the event-related scenarios with 1, 3, or 5 s yield the best results with regards to identifiability, though identifiability decreases for the modulatory inputs “Attention” and “Motion” in the 1 s scenario (mCI = 0.32 for an epoch duration of 1 s and mCI = 0.22 for 3 s). Thus, short to intermediate epoch durations rather than tenfold the TR of the original study, seem to yield full identifiability. Nevertheless, all parameters were equally identifiable in the original settings (Figure 2), though with rather large CIs.

#### Variation of SNR

**Figure 5:**
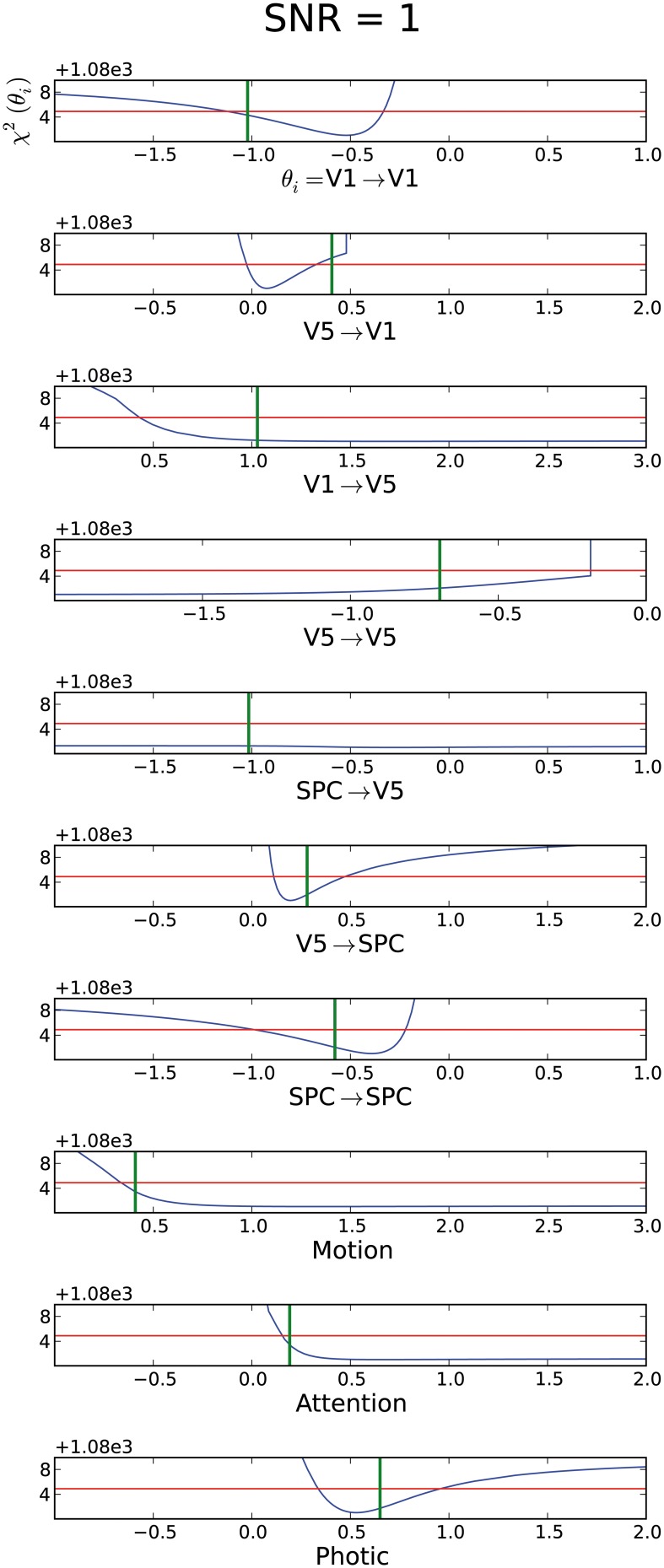
**Assessment of data with an SNR=1, where mCI = ∞**. For explanation of the graphs see Figure 2.

To investigate the influence of different SNR on identifiability, we also assessed an SNR of 1 within the original imaging specifications. From Figure 5 it is apparent that with a high noise level many parameters become practically non-identifiable and hence the mean CI is mCI = ∞. Thus, for more realistic SNR, reaching overall identifiability seems to be more difficult. Interestingly, again the connections leading to V5 and the modulatory inputs “motion” and “attention” are not identifiable. Whereas most of these parameters are still practically non-identifiable and only have a lower or upper bound, the connection from SPC to V5 exhibits a flat profile likelihood, which comprises zero. Therefore, no information is gained about this parameter. The connection from V5 to V1 is consistent with zero, even though it should be different from zero based on its real value (green line in Figure 5). Additionally, the CI does not include the real parameter value. Thus, even with a finite CI, no proper conclusion on the existence of this connection can be drawn. Hence, with a SNR of 1, many parameter values cannot be identified unambiguously as many parameters are non-identifiable or their CIs exclude the real parameter value. However, for parameters which possess a lower or upper CI bound, the parameters exist and the sign of their connection strength can unambiguously be determined.

### Results from attention to motion “backward” model

**Figure 6:**
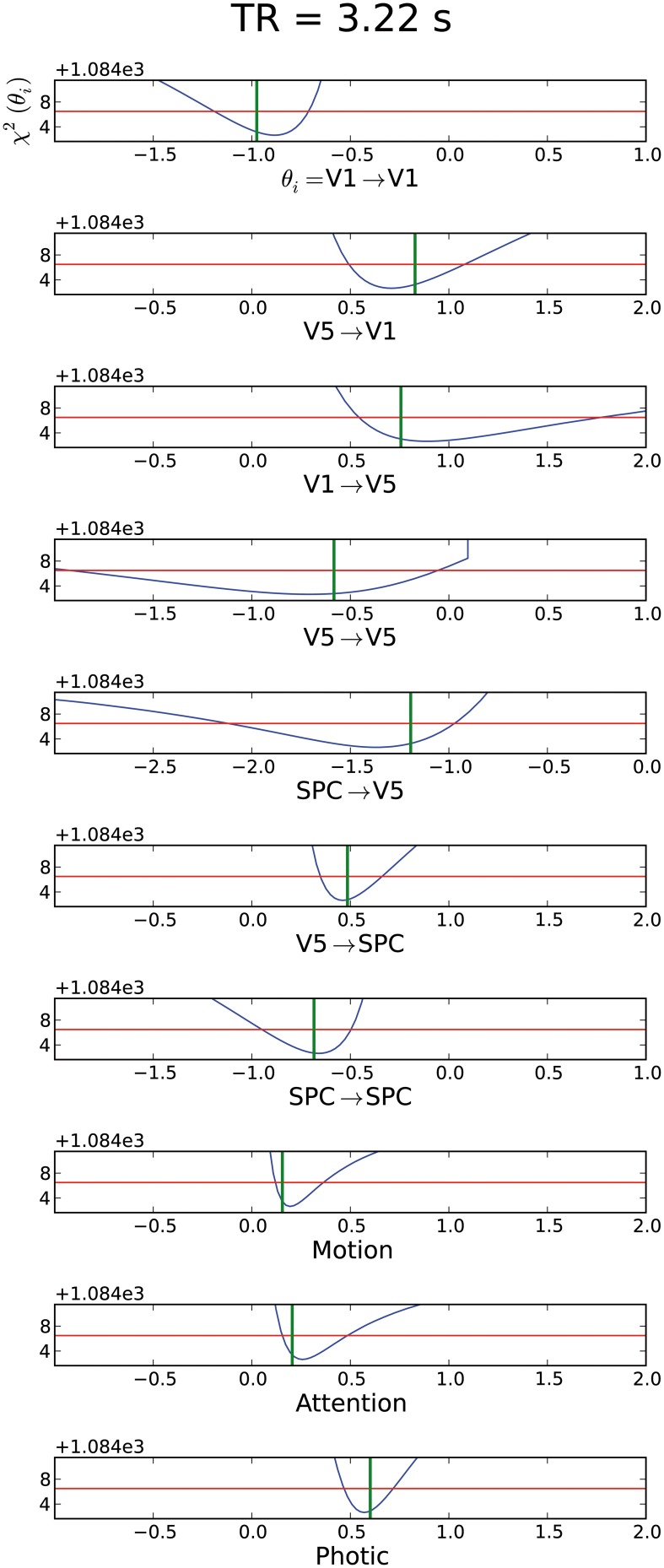
**Assessment of the data for the backward model with original settings for TR, epoch duration and session duration with mCI = 0.65**. For further explanation of the graphs see Figure 2.

Similar to the “forward” model, all parameters of the backward model in its original specification proved identifiable, though with rather large CIs on several parameter values as well (mCI = 0.65 for the backward model and mCI = 0.61 for the forward model). As the effect of manipulating TR, session and epoch duration were comparable to those on the forward model, we will only present the result of the backward model with original imaging settings (Figure 6) and provide all other profile likelihood plots of the backward model in Supplementary Material (Supplement 2).

**Figure 7:**
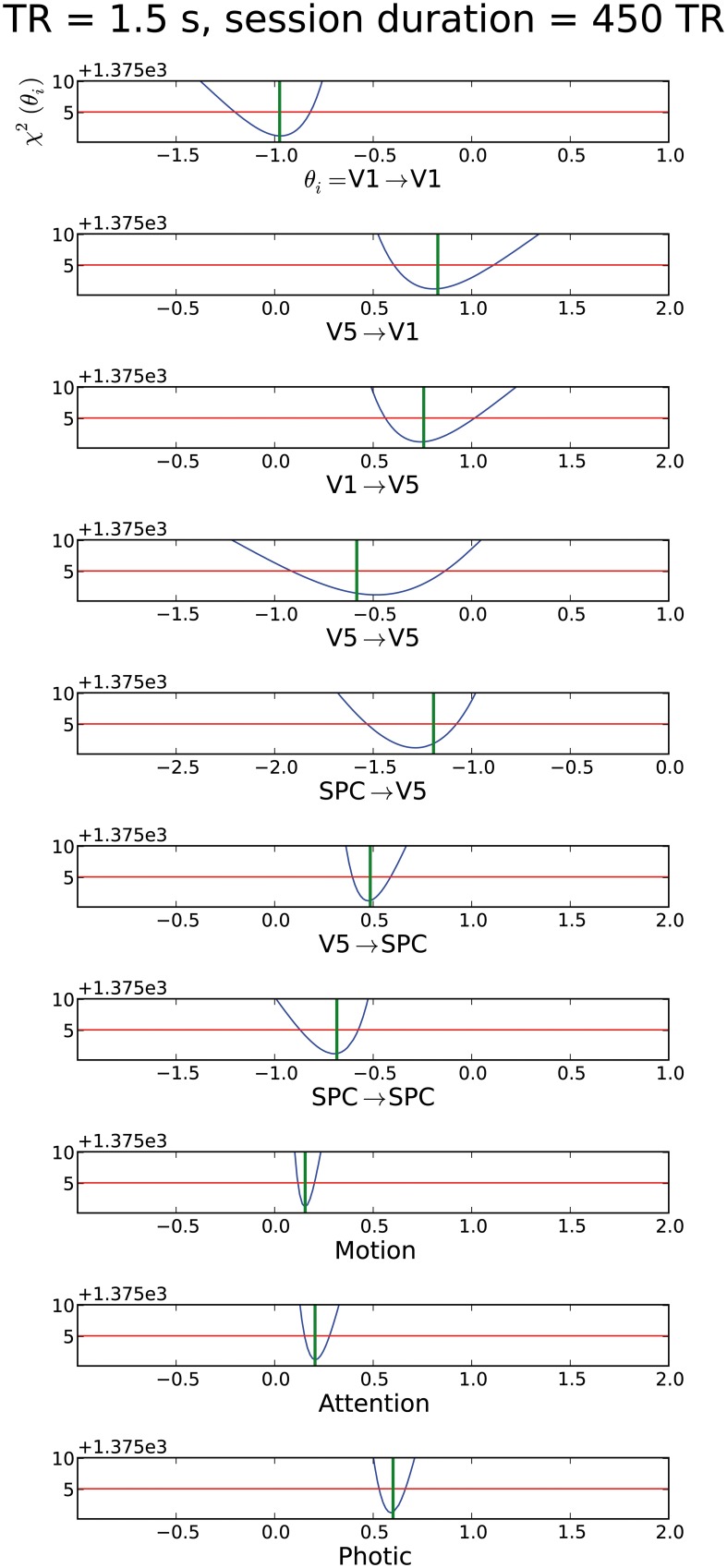
**Optimizing experimental design by manipulating two imaging specifications (here TR and session duration) at the same time, where mCI = 0.32**. For further explanation of the graphs see Figure 2.

Importantly, one might want to manipulate more than one acquisition parameter at the same time when adjusting data acquisition toward full identifiability. A decrease in TR combined with a moderate increase in session duration, i.e., the acquisition of additional 90 volumes, systematically improved parameter identifiability of the forward as well as the backward model (result for the backward model shown in Figure 7), such that a precise parameter estimation should be warranted when acquiring real fMRI data with these settings.

## Discussion

Taking the freely available “attention to motion” dataset as an example, we demonstrated an approach that permits improvements in fMRI data acquisition to ensure identifiability of all DCM parameters based on the data, and a simplified DCM model without priors. The presented DCMident toolbox operates on the profile likelihood to provide at a glance statements about parameter identifiability. Hence, we addressed the question if the data contains enough information to reliably estimate parameters that might be of interest for a specific research question or relies on Bayesian priors to restrict parameter estimates.

### Model identifiability

In two three region DCMs applied to the “attention to motion” dataset with its original MR sequence specifications, all parameters were identifiable for a high SNR of $\sqrt{\mathrm{10}}$ but often with broad CIs. The parameters of the ***B***-Matrix had much narrower CIs than those of the ***A***-Matrix. This is due to the additional information provided by the exogenous modulations “Motion” and “Attention,” specifying when experimental modulations are present or absent.

Regarding the ***A***-Matrix, CIs were widest for the connection strength between V1 and V5, V5 and V5, and SPC and V5, such that all connections leading to V5 were estimated least precisely (see original experimental specifications in TR = 3.22 s subplot in Figure 2). This can be further elucidated by assessing the number of connections or parameters (Figure 1), which have to be estimated from the time series of each node. Following this reasoning, parameter estimations from the time series of V1 are worse than the estimates from the time series of SPC, as there are three parameters to be estimated compared to two. The same rule applies to the “backward” model where only the input to region 2 (V5) changes but five parameters still have to be estimated from the respective time series. Therefore, if there are one or more densely interconnected regions in a DCM, e.g., a “central node” such as V5 in the current example, which is connected to most of the other regions, we recommend to carefully pay attention to its identifiability. When encountering practical non-identifiabilities, TR, session or epoch durations can be altered to obtain finite confidence intervals (see below). This recommendation is independent from further biological factors relating e.g., to the size and anatomical location of a node, which can additionally complicate parameter identifiability but are beyond the scope of this study. Recent methodological developments allow the inversion of DCMs with a high number of nodes (Seghier and Friston, 2013). Our framework might be an ideal preprocessing step to such endeavors by providing a heuristic as to whether densely connected nodes might follow the priors in the SPM–DCM framework when they are practically non-identifiable solely based on information in the data. Taken together, our approach can be seen as a beneficial addition to the existing SPM–DCM framework. It can variably be applied as a preprocessing step to check identifiability before conducting DCM analyses in SPM and can also be connected to recent additions to DCM.

Besides the practical non-identifiability mentioned in Section Identifiability and Non-Identifiability, additional structural non-identifiability can occur (Raue et al., 2009, 2010). For structural non-identifiable parameters, relations to other parameters can be found such that these related parameters could compensate completely for the influence of the non-identifiable parameter. Thus, structural non-identifiabilities would display themselves as flat lines without a minimum in the profile likelihood. They can occur when not all state variables are observed. In this case, not enough information exists to uniquely determine the parameter from the data. In our work, structural non-identifiabilities did not occur as we used a completely determined canonical hemodynamic response function for the sake of computation speed. Nevertheless, the presented toolbox could be modified by including the more complex balloon model (Obata et al., 2004; Stephan et al., 2007b) to fully reflect the DCM framework. However, using the more complex balloon model might potentially lead to structural non-identifiabilities, as the hemodynamic state variables and the exact onset of the hemodynamic response are not measured. This should be considered when modifying the presented framework toward an implementation of a more complex region specific balloon model.

In summary, variations of all investigated experimental design and imaging specifications had an effect on parameter identifiability (Figures 2–4). First, increasing session duration improved identifiability but not considerably beyond 540 times the TR and for this noise realization it even got slightly worse for 540 times the TR compared to 450. Hence, to ensure study participants' well-being and prevent motion artifacts, an extension of session duration to increase parameter identifiability should be carefully balanced.

Second, it is evident that decreasing TR and thereby increasing the sampling rate improves parameter identifiability, as the information for the parameter estimation is mainly gathered from the slope and height of the rise of activity due to exogenous inputs, and by increasing the sampling rate, more information on the shape of this slope is gathered. It has been a trend to shorten TR in fMRI research for several years and various reasons, see e.g., (Feinberg and Yacoub, 2012; Feinberg and Setsompop, 2013; Jacobs et al., 2014). Along this line of thought, we would recommend to reduce the number of slices and thereby TR whenever adequate for the research question at hand. This agrees with the findings from Witt and Meyerand (2009) who analyzed the influence of TR on parameter estimation. According to their analysis, decreasing TR improved parameter estimation. Hence, combining ours and their results shows that a decreased TR improves both the accuracy with which the parameter is identified and at the same time decreases confidence intervals. Of note, a decrease in TR to gain increased identifiability should lie within certain bounds, as a very short TR can substantially decrease SNR and thereby reduce identifiability (Feinberg and Setsompop, 2013). In such scenarios, manipulation of echo time (TE) might be beneficial (Krüger and Glover, 2001). As shown in the results section, TR within a range as commonly used in fMRI studies with EPI sequences, yielded sufficient identifiability.

Third, for the high SNR of $\sqrt{\mathrm{10}}$ that was chosen here, the short epoch durations analogous to event-related designs led to narrower CIs for the parameters. As the absolute number of stimuli was kept constant by keeping the distance between the stimuli constant and solely changing the length of the stimuli, this is a pure effect of epoch duration. In case of short epoch durations, the peak of the BOLD response is clearly defined and leads to well-determined parameters. However, if the epoch duration is too short (~ 1 s), the modulatory strengths *b_ij_* are estimated worse, as the switching-on and -off of a stimulus cannot be recognized (Heckman et al., 2007; De Zwart et al., 2009). This becomes apparent in the “Attention” and “Motion” results for the forward model and 1 s epoch duration (Figure 4) and is even more extreme for the “Attention” modulation on the feedback connection in the backward model Supplementary Material (Supplement 2). For long stimuli, the peak rests on a high level of activation. Therefore, parameter sets which would lead to a slightly decayed arrival at the peak would be accepted as a reasonable fit, too. Therefore, the CIs are broader for longer epoch durations.

This mechanism changes with decreasing SNR as the increasing noise impairs the detection of BOLD responses to short stimuli (Friston et al., 1999; Henson, 2007).

Correspondingly, a low SNR of 1 and epoch durations of 32.2 s together with the long original TR lead to a considerable fraction of practically non-identifiable parameters (Figure 5). As most parameters had a lower or upper bound, it is still possible to state if the respective parameter is positive or negative, which might suffice to answer certain parameter-specific research questions, e.g., relating to the question of increases or decreases in connectivity between two nodes. Limitedly, the exact amount, i.e., parameter value, of increase or decrease in connectivity cannot reliably be assessed when a parameter is practically non-identifiable. Consequently, in the case of lower SNR, the desired information can best be gained with intermediate epoch durations, which corresponds to the results by Daunizeau and colleagues from the SPM–DCM framework (Daunizeau et al., 2011b). When examining the influence of epoch duration on the model discriminability, Daunizeau et al. (2011b) found that depending on the discrimination feature between the models, an epoch duration between 8 and 16 s is optimal. In our results obtained with DCMident, an epoch duration of 8–10 s still was sufficient for parameter identifiability as well. Importantly, as there is a tight link of SNR and session duration (Murphy et al., 2007), our findings on the influence of the latter on identifiability can as well be interpreted with regards to SNR. Another scanning parameter directly influencing SNR is spatial resolution, as larger voxel size, i.e., lower resolution, enhances SNR. Although not comprised in our simulation, a trade-off on spatial resolution could lead to improved identifiability as well. Finally, the strong improvements when simultaneously decreasing TR while slightly increasing session duration (Figure 7) are of great relevance to clinical studies (Seghier et al., 2010), in which strain on participants and data acquisition duration have to be balanced.

## Conclusion on the DCMident toolbox

The DCMident toolbox presented in the current report provides solutions to ensure DCM parameter identifiability based on fMRI acquisition specifications. Identifiability is tested using the profile likelihood computed for each parameter based on a DCM with a canonical HRF and no Bayesian priors. This allows the user to judge the extent to which specific model parameters are more likely to reflect the priors than the underlying input data. Analogous to the successful application of the profile likelihood in systems biology modeling, our approach provides profile likelihood plots that are easy to interpret. At a glance, identifiability or non-identifiability as well as confidence intervals of the parameter value are depicted in the plots. When non-identifiabilities are detected, modifications of sampling rate, duration of data acquisition, stimulus frequency and noise reduction can restore identifiability. This is especially important if inference on specific model parameters is of interest. To encourage the use of sophisticated network analysis methods such as DCM for fMRI data, the DCMident toolbox will be available to interested researchers upon request from the authors as a Python-based (http://www.python.org/) toolbox package with a graphical user interface.

### Conflict of interest statement

The authors declare that the research was conducted in the absence of any commercial or financial relationships that could be construed as a potential conflict of interest.
