# Supplementary material for: Assessing parameter identifiability for dynamic causal modeling of fMRI data
Source: Front Neurosci. 2015 Feb 20;9:43. doi: 10.3389/fnins.2015.00043 (PMC4335185; doi:10.3389/fnins.2015.00043)

Supplement 1: Additional profile likelihood plots for the ‚forward‘ model

1. **TR**


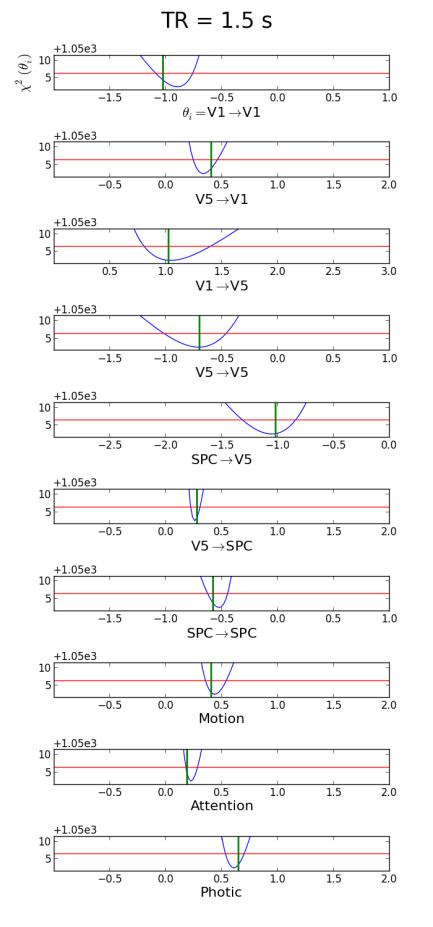

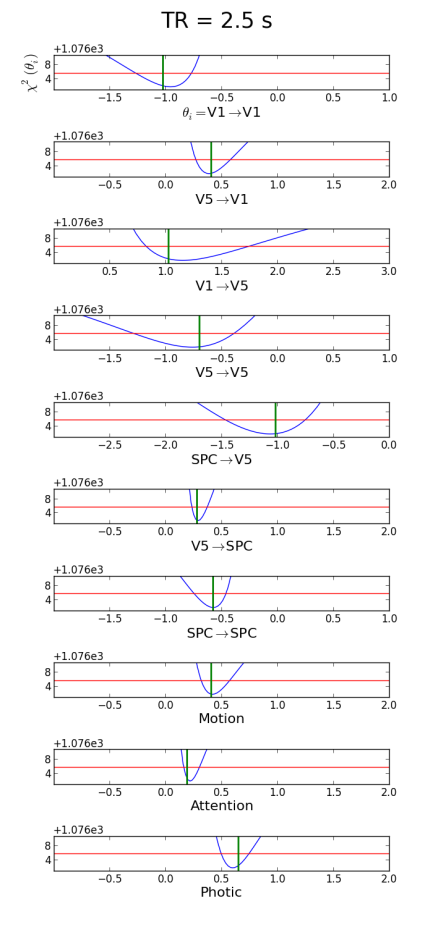

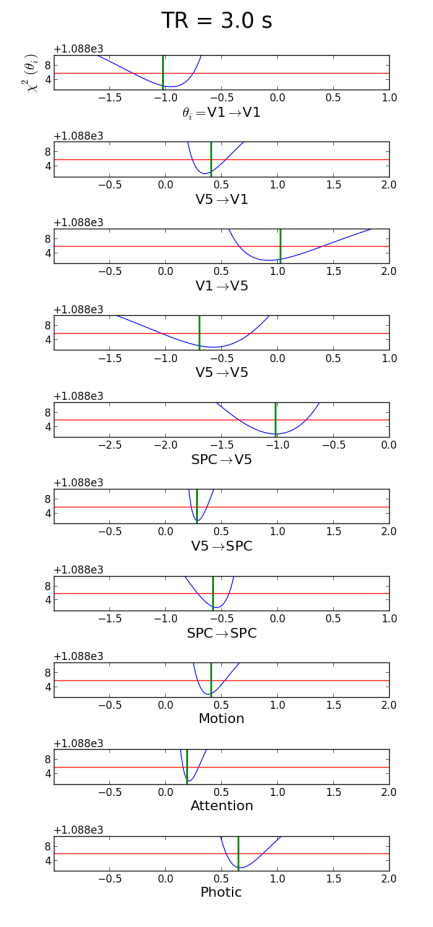


1. **Session duration**


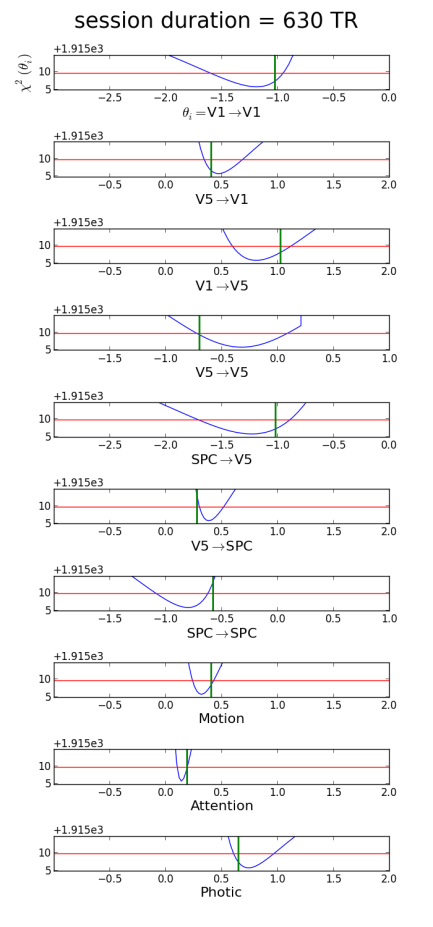

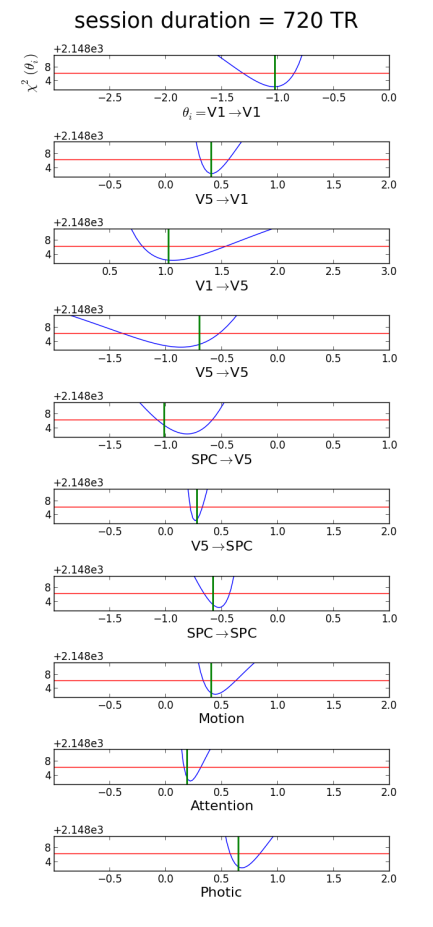


1. **Epoch duration**


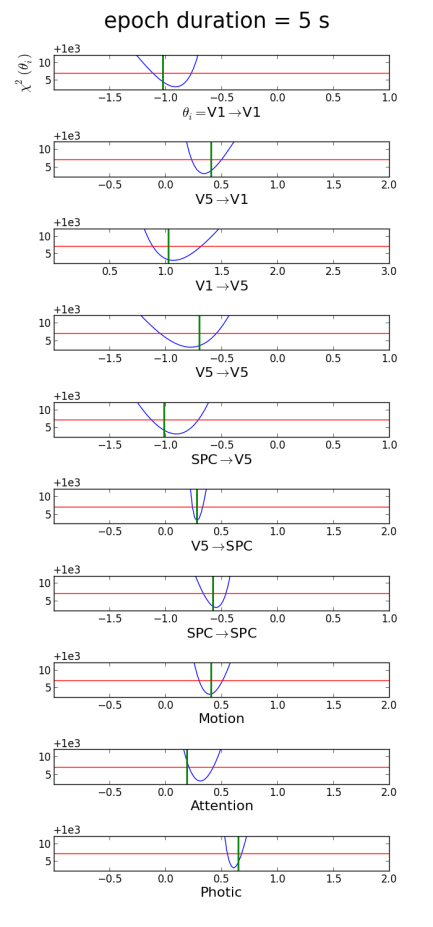

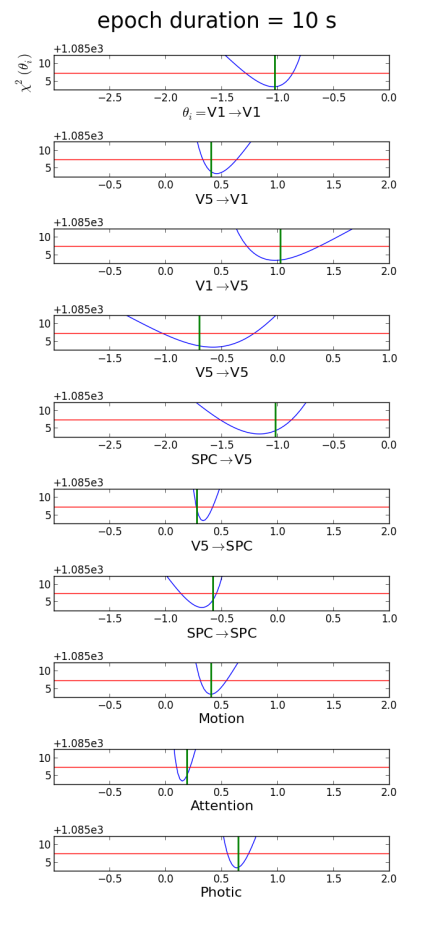

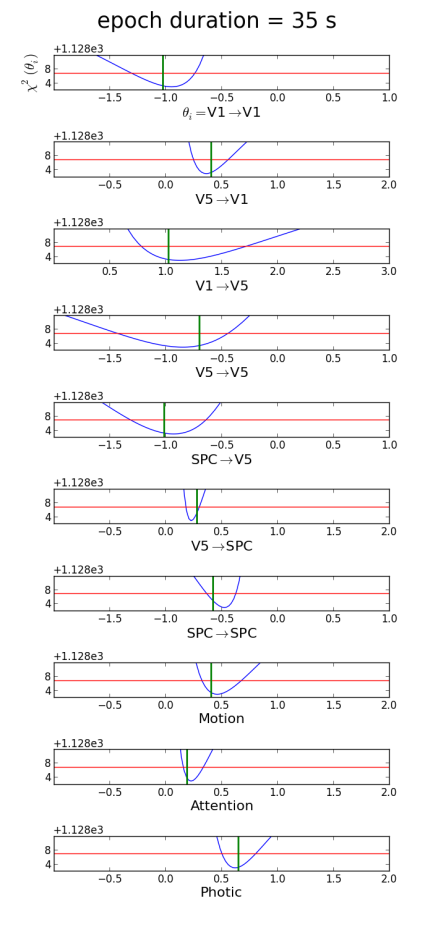

Supplement: Supplementary file 1 [file DataSheet1.DOCX]
