# Supplementary material for: Assessing parameter identifiability for dynamic causal modeling of fMRI data
Source: Front Neurosci. 2015 Feb 20;9:43. doi: 10.3389/fnins.2015.00043 (PMC4335185; doi:10.3389/fnins.2015.00043)

Supplement 2: Additional profile likelihood plots for the ‚backward‘ model

1. **TR**


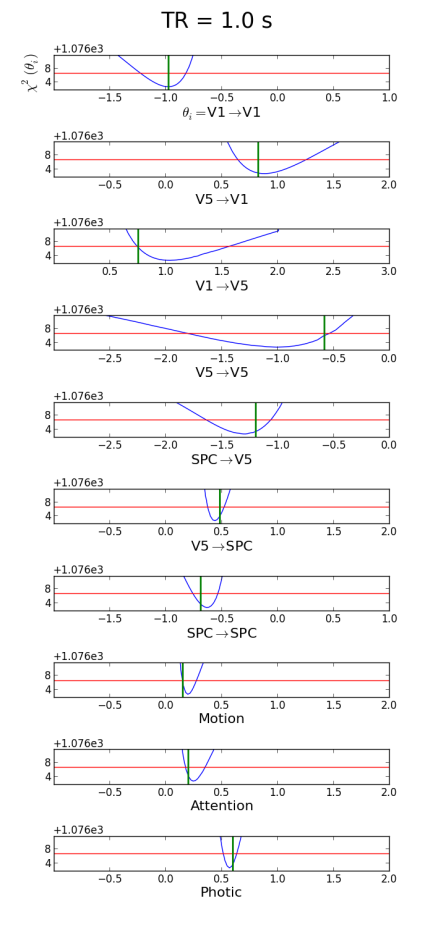

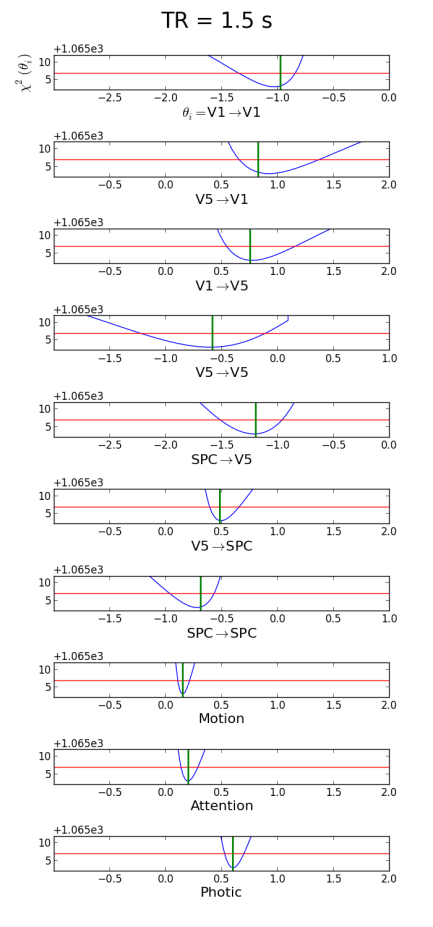

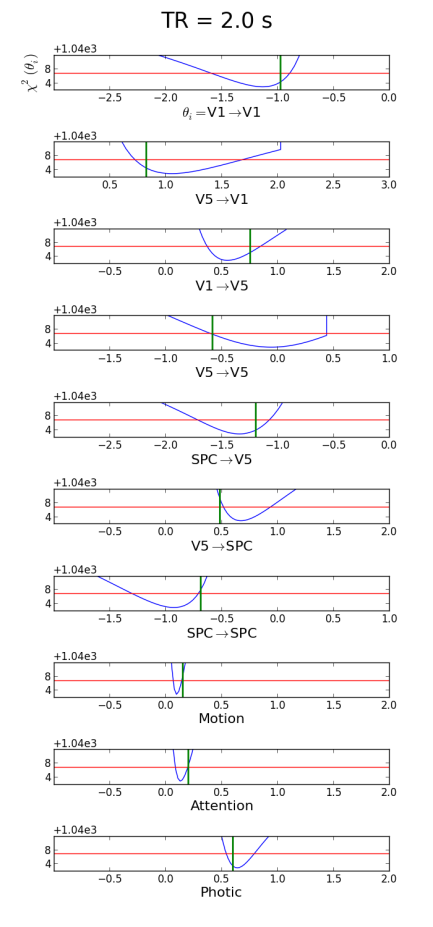

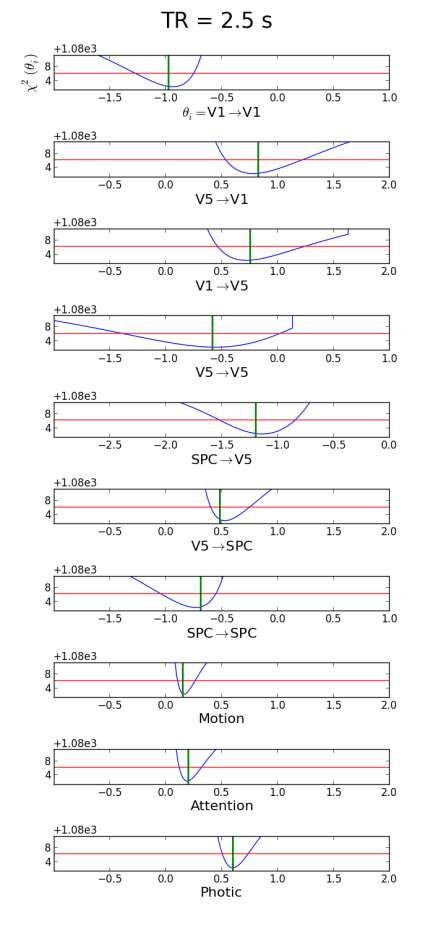

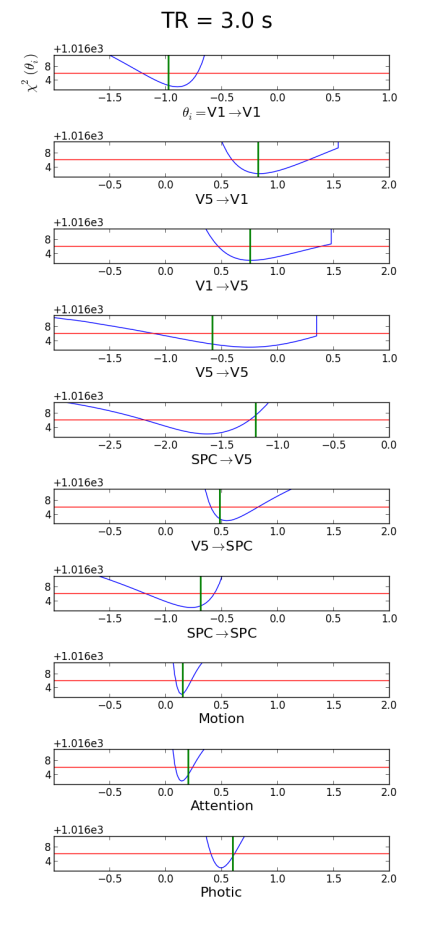


1. **Session duration**


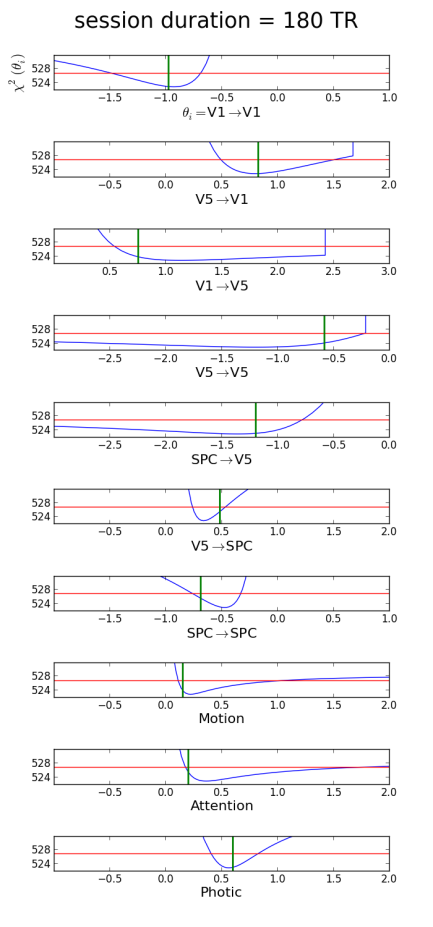

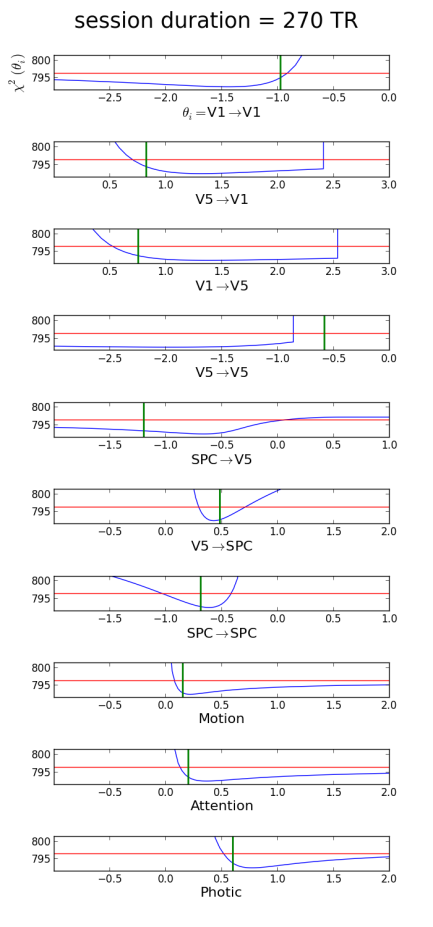

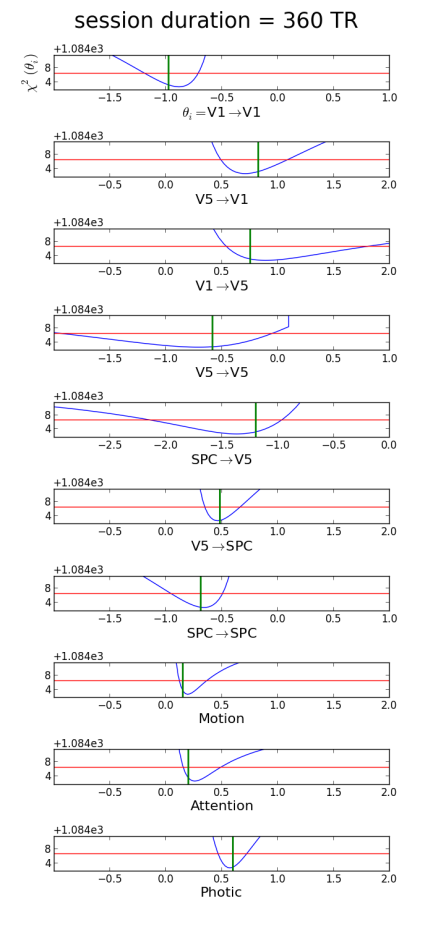

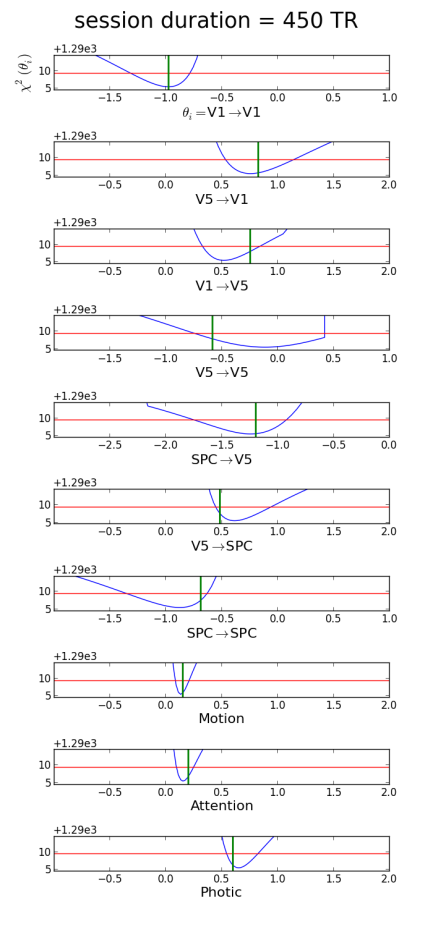


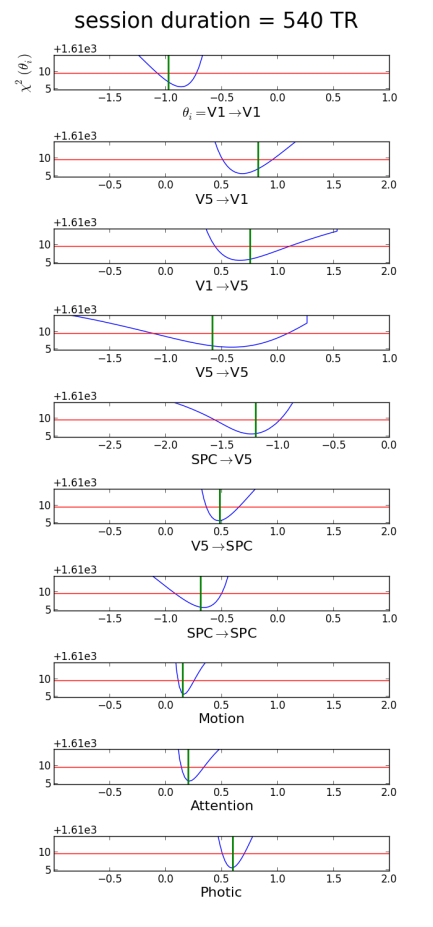

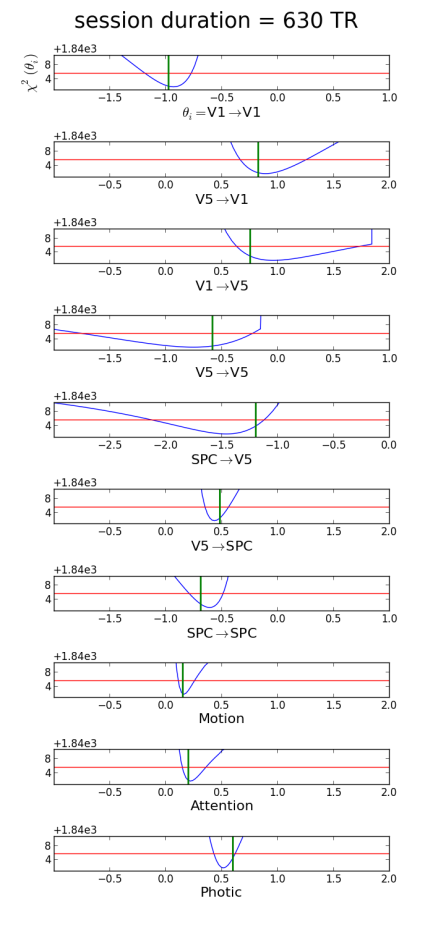

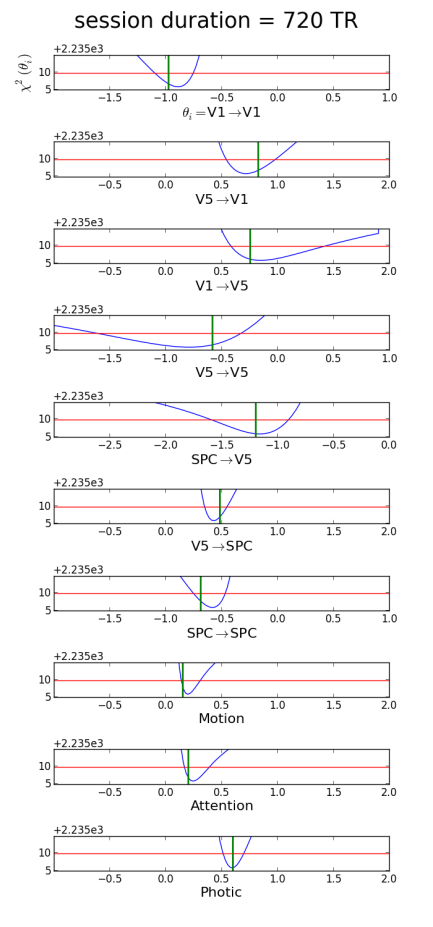


1. **Epoch duration**


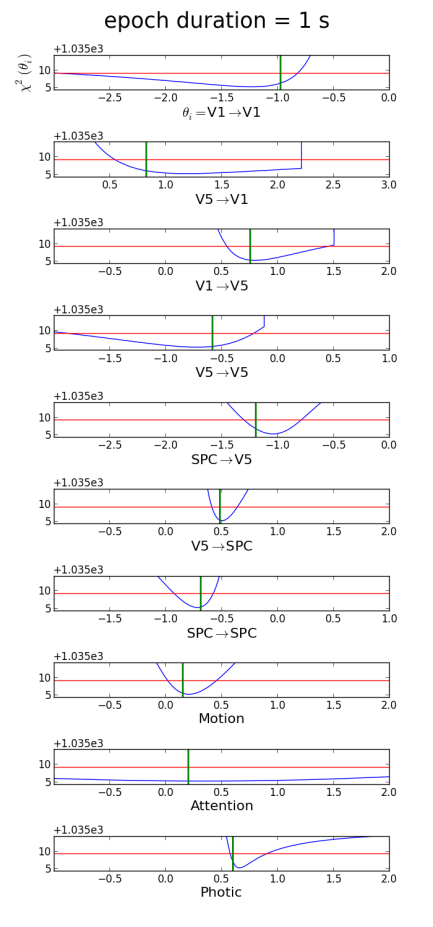

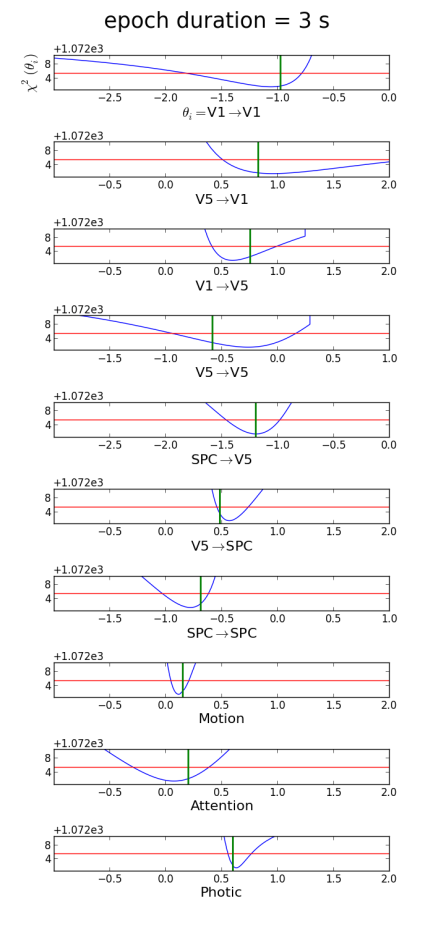

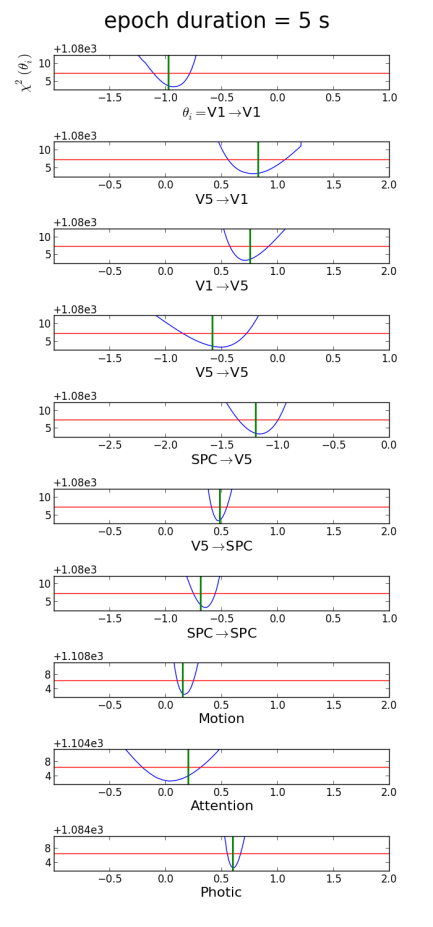

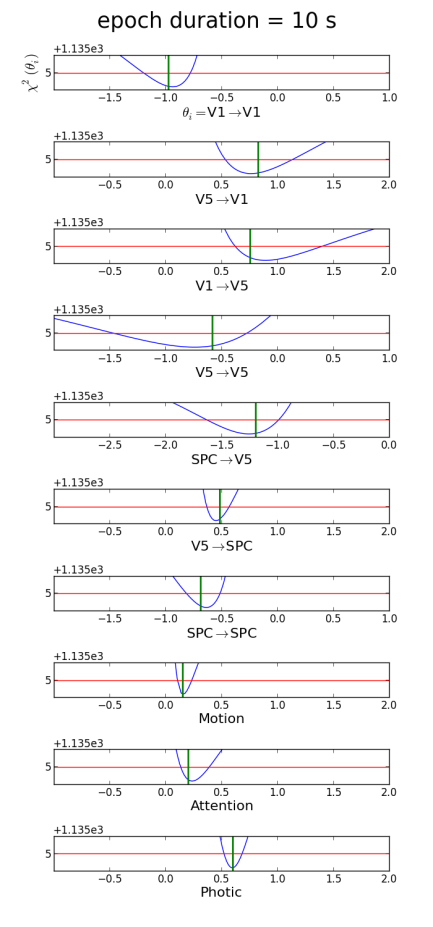

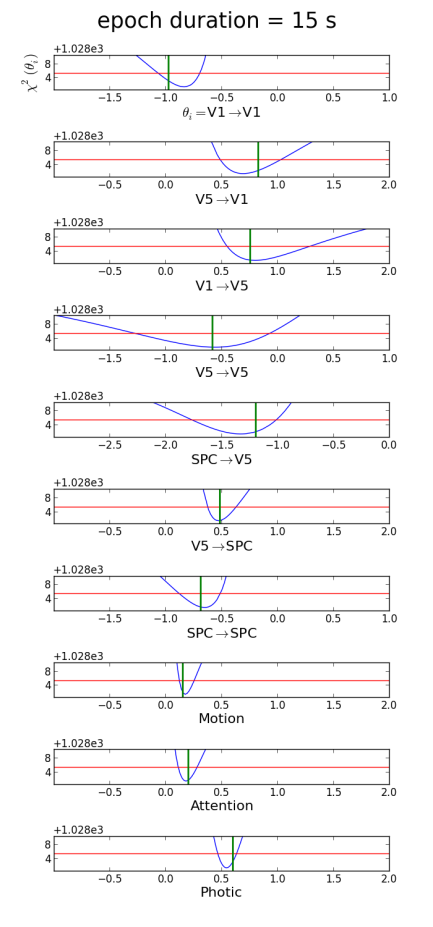


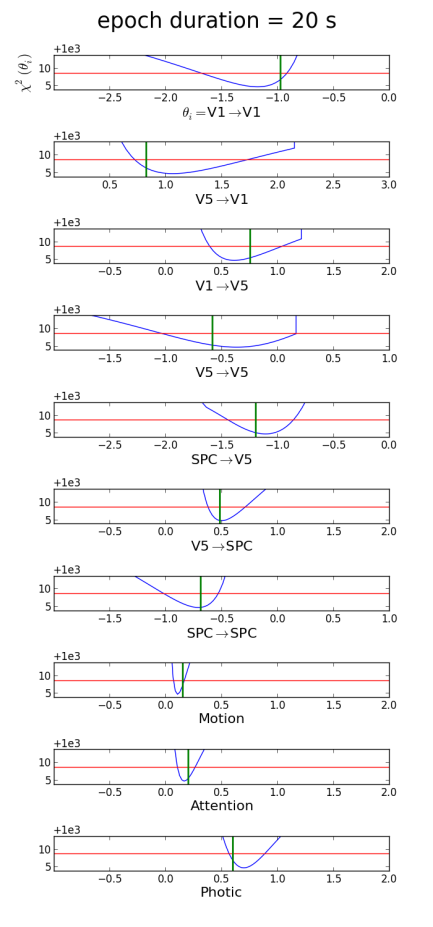

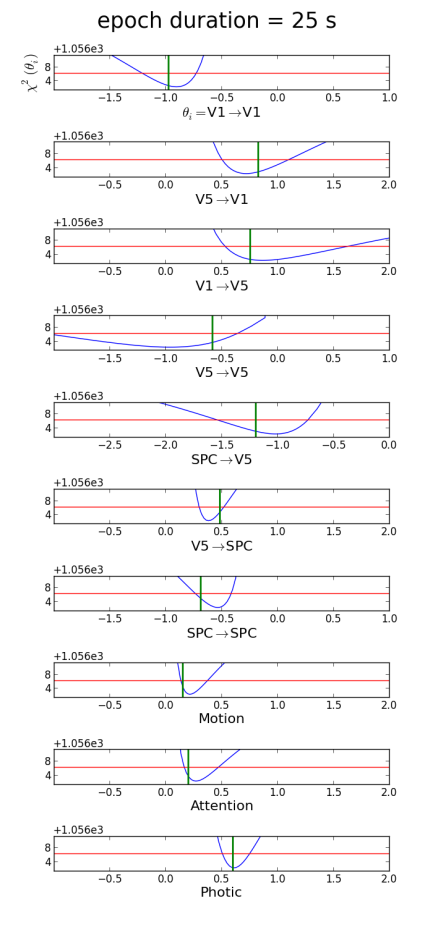

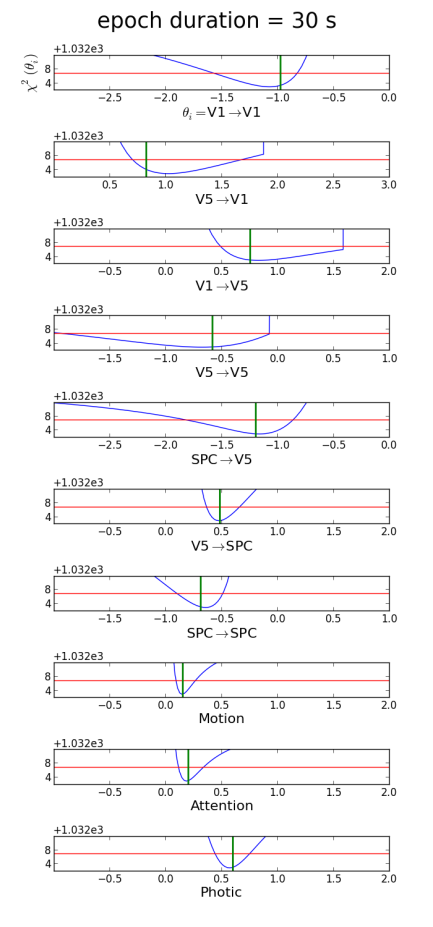

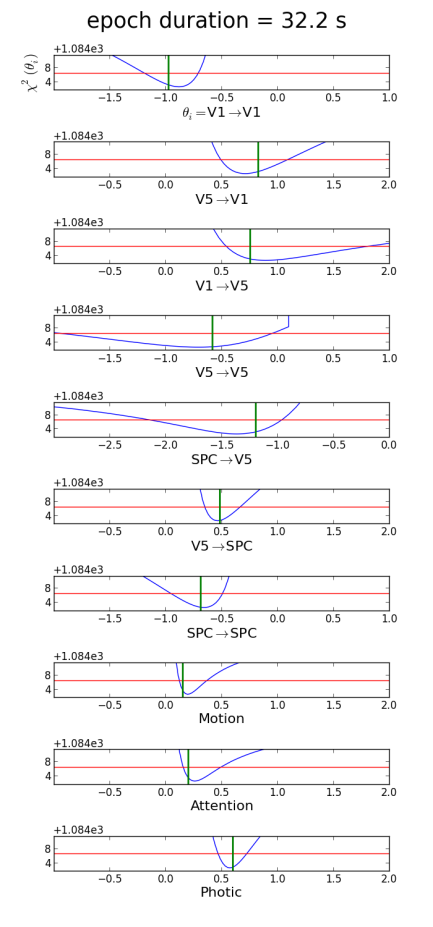

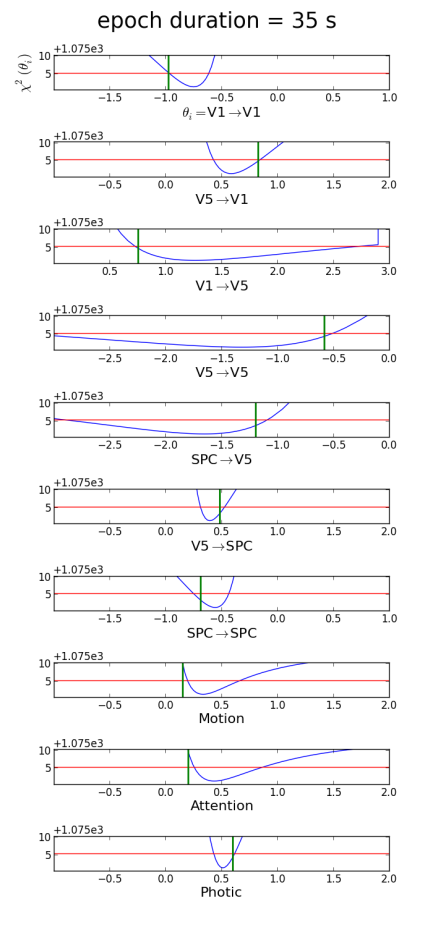

Supplement: Supplementary file 2 [file DataSheet2.DOCX]
